# Nitric Oxide and Hydrogen Sulfide Crosstalk in Plants: Redox Regulation, Stress Adaptation, and Emerging Applications

**DOI:** 10.3390/ijms27114962

**Published:** 2026-05-30

**Authors:** Roberta A. dos Reis, Amedea B. Seabra, Cecília Brilhante Aragão, Morgana Halfeld, Renan S. Nunes, Rodrigo Rodriguez, Adalberto Benavides-Mendoza, Olga Rubilar, Gonzalo R. Tortella

**Affiliations:** 1 Center for Natural and Human Sciences, Federal University of ABC (UFABC), Santo André 09210-580, SP, Brazil; roberta.reis@ufabc.edu.br (R.A.d.R.); cecilia.brilhante@ufabc.edu.br (C.B.A.); morgana.halfeld@aluno.ufabc.edu.br (M.H.); r.nunes@ufabc.edu.br (R.S.N.); 2 Instituto de Ciencias Aplicadas, Facultad de Ingeniería, Universidad Autónoma de Chile, Temuco 4810101, Chile; rodrigo.rodriguez@uautonoma.cl; 3 Department of Horticulture, Antonio Narro Autonomous Agrarian University, Calzada Antonio Narro 1923, Saltillo 25315, Mexico; adalberto.benavides@uaaan.edu.mx; 4 Conacyt Laboratory of Plant Ecophysiology and Food Safety (LANCEVSA), Antonio Narro Autonomous Agrarian University, Saltillo 25315, Mexico; 5 Centro de Excelencia en Investigación Biotecnológica Aplicada al Medio Ambiente (CIBAMA), Facultad de Ingeniería y Ciencias, Universidad de La Frontera, Temuco 4811230, Chile; olga.rubilar@ufrontera.cl; 6 Departamento de Ingeniería Química, Facultad de Ingeniería y Ciencias, Universidad de La Frontera, Temuco 4811230, Chile

**Keywords:** plant gasotransmitters, metabolite crosstalk, redox signaling, oxidative stress, nanomaterials, nanodelivery, sustainable agriculture, plant resilience

## Abstract

Nitric oxide (NO) and hydrogen sulfide (H_2_S) are key gasotransmitters that regulate multiple aspects of plant growth, development, and stress adaptation. Although their individual signaling pathways have been extensively investigated, the integrated mechanisms underlying NO–H_2_S crosstalk and its potential agronomic applications remain unclear. This review summarizes current advances in understanding the biochemical interplay between NO and H_2_S in plants, emphasizing their synergistic roles in redox regulation, antioxidant activation, ion homeostasis, and photosynthetic protection under abiotic and biotic stresses. Special attention has been given to recent progress in nanotechnology-based delivery systems that enable the controlled, localized, and sustained release of gasotransmitters, thereby improving bioavailability and minimizing environmental losses. Studies on foliar, seed, and nutrient-solution applications have demonstrated that combined NO/H_2_S treatments increase stress tolerance by activating the ascorbate–glutathione (AsA–GSH) cycle, reducing the expression of oxidative markers such as hydrogen peroxide (H_2_O_2_) and malondialdehyde (MDA), and improving both short-term (Fv/Fm, antioxidant enzyme activity) and long-term (biomass, SPAD index, yield) physiological outcomes. By integrating molecular insights with applied strategies, this review outlines the emerging potential of NO–H_2_S signaling as a sustainable tool for crop management in the context of climate change and food security.

## 1. Introduction

Gasotransmitters (GTs) are small gaseous molecules synthesized by living organisms that perform critical signaling functions. Because they can traverse cellular membranes and function independently of specific receptors, GTs are often enzymatically generated, bind distinct cellular targets, and interact with other signaling mediators [1]. The predominant plant gasotransmitters include nitric oxide (NO), carbon monoxide (CO), and hydrogen sulfide (H_2_S). Moreover, hydrogen (H_2_), methane (CH_4_), and the gaseous phytohormone ethylene (C_2_H_4_) have recently been recognized as gasotransmitters. However, their roles in plants do not always align with those of conventional GTs, and they remain underexplored [2].

GTs play diverse roles in plant biology, including cell cycle regulation, cytoskeleton formation and function, seed germination, de-etiolation, rhizogenesis, senescence, and interactions with symbionts and pathogens [1]. They are particularly crucial for enabling plant adaptation to adverse environmental conditions, with plant-derived GTs increasing in response to various abiotic stresses. Abiotic stress in plants refers to the challenges and damage caused by nonliving environmental factors, such as extreme temperatures, drought, salinity, and heavy metals. These stresses can significantly affect plant growth and productivity, leading to cellular damage and disruption of physiological processes [2]. To combat these stresses, plants have evolved complex regulatory mechanisms, including the production of reactive oxygen species (ROS) as signaling molecules.

**Figure 1:**
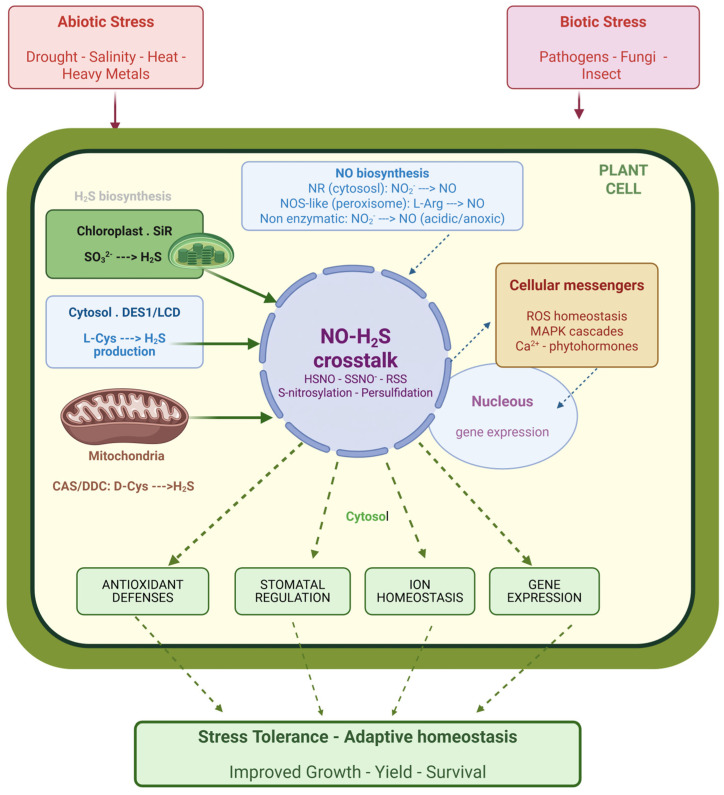
Integrative overview of NO and H_2_S signaling within plant cells in response to abiotic and biotic stress. Abiotic stressors (drought, salinity, heat, heavy metals, and oxidative damage) and biotic stressors (pathogens, fungi, insects, and herbivory) trigger the endogenous production of gasotransmitters via distinct enzymatic pathways. Nitric oxide (NO) is generated primarily via nitrate reductase (NR) and nitric oxide synthase-like (NOS-like) activity, as well as through nonenzymatic pathways. Hydrogen sulfide (H_2_S) is produced in multiple subcellular compartments: in the chloroplast by sulfite reductase (SiR), in the cytosol by L-cysteine desulfhydrase 1 (DES1) and L-cysteine desulfhydrase (LCD), and in the mitochondria by cyanoalanine synthase (CAS) and D-cysteine desulfhydrase (DDC). At the biochemical level, NO and H_2_S interact directly to generate hybrid reactive intermediates, including thionitrous acid (HSNO), nitrosopersulfide (SSNO^−^), and reactive sulfur species (RSS). At the post-translational level, both molecules can competitively or cooperatively modify cysteine residues on shared protein targets via S-nitrosylation (NO) and persulfidation (H_2_S), altering protein activity and function. Downstream convergence on reactive oxygen species (ROS) homeostasis, mitogen-activated protein kinase (MAPK) cascades, calcium (Ca^2+^) signaling, and phytohormone networks coordinates four major adaptive responses: antioxidant defense activation, stomatal regulation, ion homeostasis, and stress-responsive gene expression. The integration of these responses at the cellular level ultimately results in stress tolerance and adaptive homeostasis, thereby improving plant growth, yield, and survival under adverse conditions. Created in BioRender. Tortella, G. (2026) https://BioRender.com/p9j0bai, accessed on 1 May 2026.

In contrast, biotic stress arises from living organisms such as bacteria, fungi, and insects, triggering a distinct set of plant defense responses. These include activating immune responses and producing defensive compounds to ward off the biotic threat. Both abiotic and biotic stresses highlight the complex ways in which plants interact with and adapt to their environments, balancing growth with survival under various stress conditions (Figure 1). Nitric oxide (NO) and hydrogen sulfide (H_2_S) act as interdependent gasotransmitters that coordinate plant responses to abiotic stresses, including oxidative, osmotic, drought, temperature, and metalloid stresses. Their interaction modulates multiple cellular messengers, including reactive oxygen species (ROS), mitogen-activated protein kinase (MAPK) cascades, and phytohormones, thereby activating stress-responsive mechanisms. These include antioxidant enzyme induction, reduced lipoxygenase activity, abscisic acid (ABA) signaling, and enhanced control of permeation, ultimately contributing to improved stress tolerance and adaptive homeostasis in plants [3,4,5].

In addition to their roles, the mechanisms by which GTs activate stress-protective systems in plants remain to be fully elucidated. Although the understanding of GT targets remains nascent and fragmented, knowledge is accumulating. GTs maintain intricate functional interrelationships with each other, the broader plant cell signaling network, and plant hormones [1]. Notably, research on the use of gases such as O and H_2_S in agritechnology is burgeoning [6,7,8]. The dynamic interplay between H_2_S and NO underscores the complexity of plant signaling mechanisms and their significance in plant biology [1].

Despite the growing recognition of NO and H_2_S as key gasotransmitters in plant physiology, their integrated mechanisms under real agronomic conditions remain underexplored, particularly regarding NO–H_2_S crosstalk and nano-enabled delivery systems. This review addresses these gaps by bridging plant physiology, biotechnology, and nanoscience to offer a timely perspective for developing resilient agricultural systems under climate stress.

While previous reviews have addressed NO or H_2_S signaling individually or in broader gasotransmitter contexts [9,10], a comprehensive and integrated framework that captures the biochemical crosstalk between these two molecules, encompassing shared redox targets, cooperative stress-response nodes, nanotechnology-enabled delivery, and real-world agronomic relevance, remains absent from the literature. Most prior work has focused on single-molecule effects under controlled laboratory conditions, addressing how NO and H_2_S interact dynamically across different stress scenarios, crop species, or application strategies. Furthermore, the potential of nanocarrier-based co-delivery systems to harness synergistic NO–H_2_S signaling in planta has not been systematically reviewed. This review fills these gaps by providing the first integrated analysis of NO–H_2_S crosstalk across molecular, physiological, and applied dimensions.

**Figure 2:**
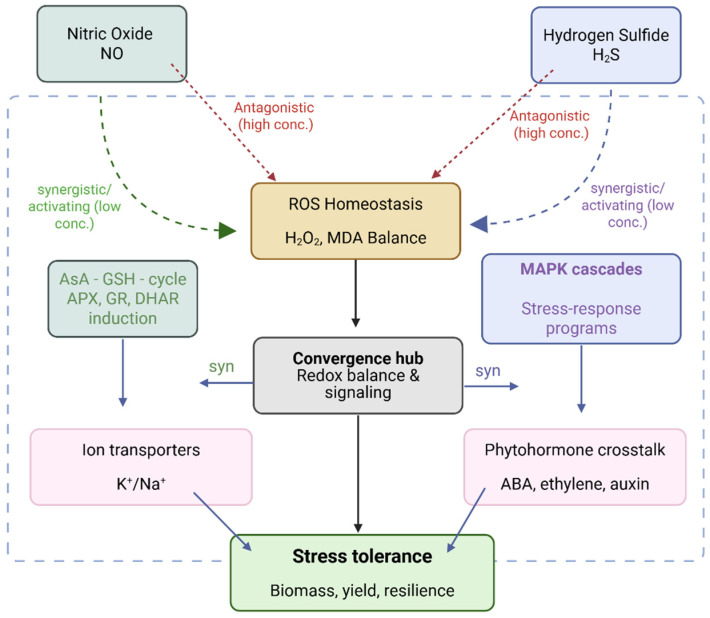
Conceptual framework of NO–H_2_S crosstalk in plant stress signaling. The diagram illustrates the five principal convergence nodes: the ascorbate–glutathione (AsA–GSH) antioxidant cycle, ROS homeostasis, MAPK signaling cascades, ion transporter regulation, and phytohormone crosstalk (ABA, ethylene, and auxin). The arrows indicate activating interactions; the blunt-ended lines indicate inhibitory interactions. Synergistic (green) and antagonistic (red) modes of action are context-dependent and vary with stress type, gasotransmitter concentration, and subcellular compartment. The dashed boundary highlights the spatial and temporal dimensions that modulate crosstalk outcomes.

To provide a conceptual anchor for this review, Figure 2 illustrates the principal nodes where NO and H_2_S signaling converge and diverge in plant cells. These nodes include (i) the ascorbate–glutathione (AsA–GSH) cycle, where both molecules cooperatively upregulate key antioxidant enzymes (APX, GR, and DHAR) to mitigate oxidative damage; (ii) ROS homeostasis, where NO and H_2_S exhibit context-dependent synergistic or antagonistic interactions depending on stress intensity and cellular redox state; (iii) MAPK cascades, which transduce combined NO–H_2_S signals into transcriptional stress-response programs; (iv) ion transporters and channel regulation, particularly K^+^/Na^+^ homeostasis under salinity stress; and (v) phytohormone crosstalk, notably with ABA, ethylene, and auxin pathways that modulate stomatal dynamics and root architecture. Critically, the mode of interaction, synergistic vs. antagonistic, is not fixed but depends on the spatial context (apoplast vs. cytosol vs. nucleus), temporal dynamics (acute vs. chronic stress), and concentration thresholds of each gasotransmitter. This spatial and temporal dimension of NO–H_2_S crosstalk represents one of the most underexplored aspects of plant gasotransmitter biology and constitutes a central focus of this review.

NO is a redox-active molecule synthesized in plant cells primarily through nitrate reductase (NR)-mediated reduction in nitrite and via NOS-like enzymatic activity from L-arginine, giving rise to reactive nitrogen compounds that mediate downstream signaling events [7,11,12,13]. At the mechanistic level, NO biosynthesis in plants operates through two principal enzymatic routes. The reductive pathway involves the two-step conversion of nitrate (NO_3_^−^) to nitrite (NO_2_^−^) by nitrate reductase (NR), followed by further reduction in nitrite to NO under low-oxygen or acidic conditions, primarily in the cytosol and apoplast [11,13]. The oxidative pathway, analogous to mammalian NOS activity, involves the oxidation of L-arginine to NO and L-citrulline via NOS-like enzymes localized in peroxisomes, chloroplasts, and mitochondria. However, the identity of a canonical plant NOS gene remains unresolved [12,14]. Additionally, non-enzymatic NO generation can occur through the spontaneous reduction of nitrite under acidic or anaerobic conditions, representing a physiologically relevant source during hypoxic stress [13].

Initially, scrutinized in animals, the importance of NO in plants, especially concerning plant immunity and plant‒pathogen interactions, has gained increasing recognition [7]. NO regulates various processes critical to plant growth, development, and adaptation to environmental stress. In model plants and economically significant crops such as rice and wheat, NO has been shown to increase seed germination, seedling growth, biomass accumulation, and yield in diverse vegetables, flowers, and fruits [7]. It effectively breaks seed dormancy and stimulates germination, playing a vital role in processes that utilize nitrogenous compounds such as nitrate and nitrite [14]. NO contribution is particularly evident in horticultural practices that employ smoke to break dormancy and stimulate the germination of specific vegetables [15,16].

Furthermore, NO is instrumental in regulating the coordinated growth of primary, lateral, and adventitious roots, with a notable influence on auxin-mediated root development, thereby shaping root architecture [11]. NO is also essential for nutrient management in horticulture, particularly for optimizing yields and profitability, as its concentration increases in plant tissues in response to changes in nutrient supply [7,11,12]. Critically, NO is vital for counteracting abiotic stressors, including salinity, drought, and extreme temperatures, and for mediating plant responses that foster growth and development under adverse conditions [7,11,13].

In conjunction, hydrogen sulfide has emerged as a significant signaling molecule in plants, particularly in the stress response. It regulates a multitude of physiological and developmental processes, mediating stress tolerance by modulating the levels of antioxidants, glutathione (GSH), osmoregulatory accumulation, cell signaling proteins, and stress-related genes [1,6,17,18]. This regulation helps balance reactive oxygen species (ROS) levels and maintain intracellular redox homeostasis [19]. H_2_S is pivotal in plant sulfur metabolism and sulfur-based signaling, mitigating the detrimental effects of environmental stresses, including drought, temperature extremes, salinity, and heavy metal stress. At the mechanistic level, H_2_S biosynthesis in plants is compartment-specific and relies on distinct enzymatic routes depending on the subcellular location. In the chloroplast, sulfite reductase (SiR) catalyzes the reduction of sulfite (SO_3_^2−^) to H_2_S as part of the primary sulfur assimilation pathway, representing the principal source of H_2_S under non-stress conditions [1,20]. In the cytosol, L-cysteine desulfhydrase 1 (DES1) and L-cysteine desulfhydrase (LCD) catalyze the desulfhydration of L-cysteine to produce H_2_S, pyruvate, and ammonia, with DES1 activity being particularly relevant during senescence and guard cell signaling [18,21]. In the mitochondria, β-cyanoalanine synthase (CAS) and D-cysteine desulfhydrase (DDC) provide an additional source of H_2_S through the metabolism of D-cysteine, a pathway whose physiological significance under stress conditions is increasingly recognized [20,22].

It primarily combats stress by modulating ROS metabolism and influencing antioxidant levels, signaling pathways, and protein activity via cysteine persulfidation. These modifications can induce long-term changes in secondary metabolite profiles and gene expression [15,20]. The increasing climate crisis, fuelled by anthropogenic activities, demands innovative approaches to safeguard horticulture. H_2_S-based treatments show promise, potentially addressing a range of challenges, including enhanced photosynthetic efficiency, improved postharvest management, increased tolerance to drought, salt stress, and temperature extremes, balanced nutrient availability, and increased heavy metal tolerance [23]. This strategy is vital for adapting to the diverse impacts of climate change, including temperature fluctuations, flooding, and increased soil salinity and contamination, thereby ensuring the sustainability and resilience of horticultural practices [6,17,18].

## 2. Interplay Between NO and H_2_S During Plant Development

### 2.1. Chemical and Molecular Basis of the NO–H_2_S Interaction

A critical yet underexplored dimension of NO–H_2_S coordination lies in their capacity to modify the same protein targets through competing or complementary post-translational modifications (PTMs). NO primarily acts via S-nitrosation, the covalent attachment of a nitroso group (–NO) to cysteine thiol residues (–SH), generating S-nitrosothiols (SNOs) that alter protein conformation, enzymatic activity, and subcellular localization [24,25]. In contrast, H_2_S drives persulfidation (also termed S-sulfhydration), converting cysteine–SH groups into persulfide species (–SSH), which generally confer protection against irreversible oxidative damage [20,26]. Crucially, these two modifications are not independent: S-nitrosated cysteines can be transiently converted to persulfide species in the presence of H_2_S, generating a dynamic post-translational modification equilibrium that fine-tunes the activity of shared target proteins, including glyceraldehyde-3-phosphate dehydrogenase (GAPDH), aquaporins, and components of the AsA–GSH cycle [21,27,28]. Furthermore, the direct chemical reaction between NO and H_2_S can produce hybrid S/N intermediates, including thionitrous acid (HSNO) and nitrosopersulfide (SSNO^−^), the latter being considerably more stable at physiological pH and being a plausible mobile transnitrosating agent capable of reaching distant protein targets [29]. This biochemical layer of interaction, operating independently of gene expression changes, represents a direct molecular interface between the two gasotransmitters and must be distinguished from their downstream convergence on shared signaling pathways [24].

**Figure 3:**
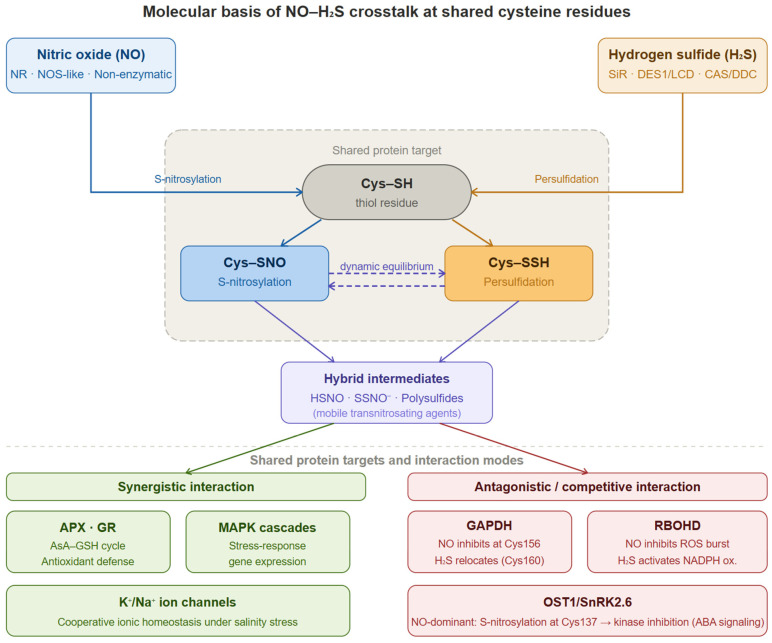
Molecular basis of NO–H_2_S crosstalk at shared cysteine residues. NO drives S-nitrosylation (Cys–SNO) while H_2_S promotes persulfidation (Cys–SSH) on shared protein cysteine residues. Both modifications exist in dynamic equilibrium and generate hybrid intermediates (HSNO, SSNO^−^, polysulfides) with independent signaling activity. The lower panel classifies the principal shared protein targets by interaction mode: synergistic (green), where both gasotransmitters cooperatively regulate APX/GR, MAPK cascades, and K^+^/Na^+^ channels; and antagonistic/competitive (red), where NO and H_2_S produce opposing functional outcomes on GAPDH, RBOHD, and OST1/SnRK2.6. In the lower panel, arrows indicate that the corresponding modification leads to the indicated functional effect.

It is conceptually important to distinguish two levels at which NO and H_2_S interact in plant cells. The first is at the direct biochemical level, where the two molecules react chemically to generate hybrid intermediates, namely, thionitrous acid (HSNO), nitrosopersulfide (SSNO^−^), and polysulfides, which themselves carry signaling activity [29]. The second is signaling-level convergence, in which NO and H_2_S, acting through separate post-translational cascades, independently regulate overlapping downstream targets, such as MAPK modules, antioxidant enzymes, and ion channels, producing synergistic or antagonistic outputs depending on the cellular context. Confounding these two levels has been a persistent source of interpretive ambiguity in the literature. For instance, the ability of H_2_S to reverse S-nitrosation on GAPDH and aquaporins may reflect either a direct chemical displacement reaction at the cysteine residue [21,27] or an indirect redox buffering effect mediated by H_2_S-driven upregulation of glutathione (GSH). Distinguishing between these mechanisms requires time-resolved proteomic approaches that can capture the sequential occupancy of individual cysteine residues under combined NO/H_2_S treatments. The contexts in which NO and H_2_S act synergistically or antagonistically, including dose-dependent, species-specific, and donor-dependent outcomes, are analyzed in detail in Section 4.3.6. This experimental frontier remains largely unexplored in plant systems. Beyond their direct biochemical interaction, the NO–H_2_S module is further regulated by upstream orchestrators, most notably melatonin, which simultaneously induces NOS-like gene expression to promote NO accumulation and activates L/D-cysteine desulfhydrase (L/D-CD) to increase endogenous H_2_S levels, forming a sequential amplification network that converges on ROS homeostasis and the AsA–GSH cycle; this hierarchical relationship is discussed in detail in Section 3 [30,31]. Table 1 summarizes the principal shared molecular targets of NO and H_2_S, their respective PTMs, and the mode of interaction in each signaling context, and these are schematically illustrated in Figure 3.

### 2.2. NO and H_2_S Across Plant Developmental Stages

**Figure 4:**
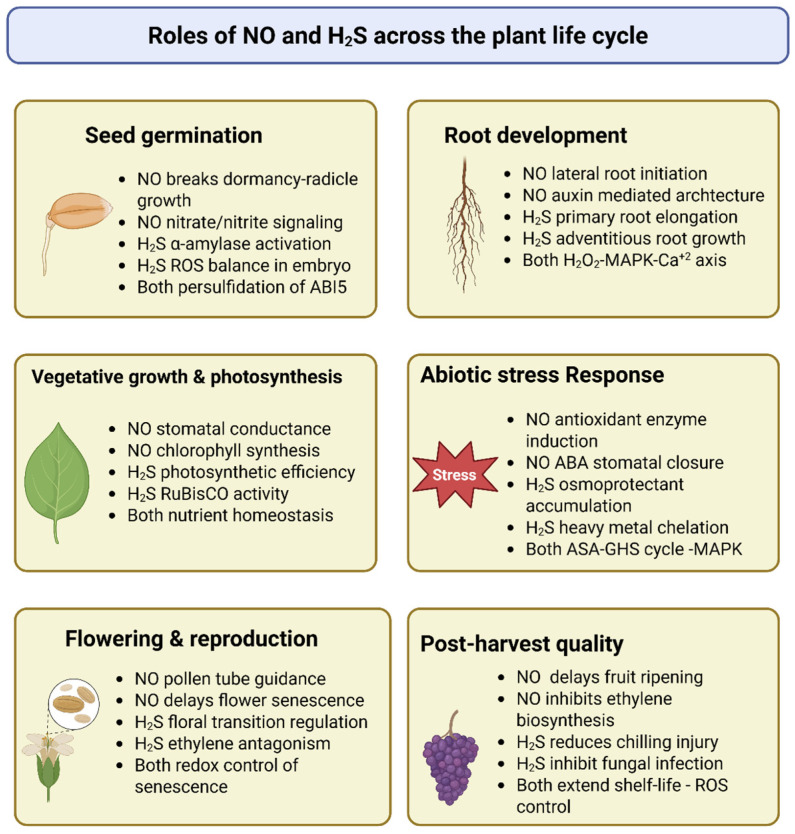
Physiological roles of nitric oxide (NO) and hydrogen sulfide (H_2_S) across the plant life cycle. Six developmental and agronomic stages are shown: seed germination, root development, vegetative growth and photosynthesis, abiotic stress response, flowering and reproduction, and postharvest quality management. At each stage, NO and H_2_S exert both independent and cooperative effects on plant physiology, which are mediated at the molecular level by post-translational modifications (PTMs) on shared cysteine residues, S-nitrosylation by NO, and persulfidation by H_2_S.

Building on the molecular framework established in Section 2.1, the cooperative and antagonistic actions of NO and H_2_S manifest across all major stages of the plant life cycle, from seed germination to postharvest management. At each developmental stage, both gasotransmitters exert stage-specific effects while converging on shared signaling nodes, ROS homeostasis, phytohormone cascades, and ion channel regulation to produce coordinated physiological responses, as illustrated in Figure 4 [21,25,26,27,28,29,30,31,32,33].

In the context of seed germination, NO and H_2_S regulate key physiological events. They interact with the thiol groups of cysteine residues in proteins, thereby affecting the functionality of molecules crucial to the germination process. Moreover, their interaction is closely linked to ROS metabolism in plant cells, which is integral to the management of environmental stress responses during seed germination. This complex biochemical interplay between NO, H_2_S, and other molecules, such as iron in proteins, highlights their vital roles in plant processes beyond germination, including photosynthesis and respiration. Understanding the mechanisms and effects of NO and H_2_S on seed germination not only deepens our knowledge of plant physiology but also opens avenues for biotechnological advancements in agriculture [26].

The interplay between NO and H_2_S is integral to root development and signaling in plants, particularly under varying environmental conditions. These gasotransmitters, along with hydrogen peroxide (H_2_O_2_), are key modulators of root system architecture and development. They operate within a complex network in which their concentration and application determine whether their effects on H_2_O_2_ signaling are synergistic or antagonistic, a crucial factor in promoting oxidative stress tolerance in roots. This involves the coordinated orchestration of components such as mitogen-activated protein kinases (MAPKs cyclins) and calcium flux [34]. Both NO and H_2_S share overlapping physiological roles and interact with other cellular oxidants, influencing cellular redox regulation. Particularly notable is the role of H_2_S in persulfidation, a reversible protein modification that targets key antioxidant enzymes and is essential for processes such as auxin signaling and ROS homeostasis. This intricate crosstalk among NO, H_2_S, and H_2_O_2_ is essential for proper development and stress response mechanisms in plant roots, highlighting the complexity and significance of these signaling molecules in plant physiology [34].

## 3. Interplay Between NO and H_2_S During Abiotic and Biotic Stress Responses

H_2_S and NO play crucial roles in plant responses to abiotic and biotic stresses, particularly through their interactions in stomatal regulation. The stomatal aperture, which is vital for balancing water loss and photosynthetic CO_2_ uptake, is influenced by H_2_S and NO signaling. H_2_S, acting as a signaling molecule, regulates stomatal movement by affecting phytohormones, ion homeostasis, and cell structural components. Its interaction with other signaling molecules, such as NO and H_2_O_2_, in guard cells is particularly significant. Exogenous H_2_S, via donors such as NaHS, promotes stomatal closure, suggesting a positive role in stomatal regulation.

Furthermore, H_2_S is involved in abscisic acid (ABA)-induced stomatal closure and promotes NO generation, with NO acting downstream. This interaction extends to regulating the dynamic balance of ROS in guard cells. A unique aspect of H_2_S function is its involvement in post-translational modification of protein cysteine residues through persulfidation, similar to S-nitrosylation by NO, as described in Section 2.1. This process indicates that H_2_S reduces cellular oxidative stress, highlighting its significant role in plant stress responses [26]. The coordination between NO and H_2_S in ABA-mediated stomatal closure illustrates how signaling-level convergence operates in a defined physiological context. ABA triggers the production of both gasotransmitters in guard cells via distinct enzymatic pathways: H_2_S is primarily generated by L-cysteine desulfhydrase (DES1).

Moreover, NO is produced downstream via nitrate reductase (NR). Evidence from *des1* knockout mutants in *Arabidopsis thaliana* has demonstrated a hierarchical relationship in which H_2_S acts upstream of NO, DES1-derived H_2_S is required for ABA-stimulated NO accumulation in guard cells, and exogenous H_2_S restores stomatal closure in *des1* mutants that otherwise fail to respond to ABA [32]. At the biochemical level, H_2_S and NO converge on shared guard cell protein targets through their respective PTMs: persulfidation of RBOHD by H_2_S increases NADPH oxidase activity and promotes ROS production [21], whereas S-nitrosation of OST1/SnRK2.6 by NO modulates its kinase activity in a manner that fine-tunes the ABA signaling output in guard cells [21].

Notably, proteomic comparisons revealed that 639 proteins in *Arabidopsis* are susceptible to both persulfidation and S-nitrosation [21], underscoring the scale at which these two PTMs compete or cooperate on shared targets. This biochemical fact goes far beyond simple additive signaling. A third regulatory layer of the NO–H_2_S signaling module involves melatonin, a pleiotropic molecule that acts as a hierarchical upstream orchestrator of both gasotransmitters rather than a simple cooperative partner [30]. Gu et al. studied salinity, drought, cold, and heat stress, particularly in combination with melatonin. NO, as a downstream signal of melatonin, regulates plant tolerance to diverse stressors, including salinity, drought, and heavy metal exposure. The regulation of the nitric oxide synthase gene by melatonin enhances NO accumulation, thereby bolstering plant resilience under stressful conditions.

Similarly, melatonin modulates the activity of enzymes involved in H_2_S production, thereby increasing H_2_S levels that help mitigate oxidative stress and environmental damage from salinity and heat. The interplay between NO and H_2_S, especially in the presence of melatonin, forms a robust defense mechanism that enhances antioxidative responses and reduces oxidative damage, thereby improving plant resistance to a variety of abiotic stresses [30].

The relationship between melatonin and the NO-H_2_S signaling module is better understood as a hierarchical regulatory interaction rather than a simple cooperative effect. Melatonin acts as an upstream orchestrator: it stimulates the upregulation of nitric oxide synthase-like (NOS-like) gene expression, thereby promoting endogenous NO accumulation, while simultaneously inducing L/D-cysteine desulfhydrase (L/D-CD) activity to increase endogenous H_2_S levels [30,31].

The above was clearly demonstrated in pepper (*Capsicum annuum*) plants under combined salt stress and iron deficiency, where melatonin-induced tolerance was abolished when either a NO scavenger (cPTIO) or an H_2_S scavenger (hypotaurine) was applied. Notably, the H_2_S effect was suppressed by both scavengers, suggesting that H_2_S acts downstream of NO within the melatonin signaling cascade [31]. On the other hand, in cucumber under salinity, melatonin-triggered H_2_S production further activated MAPK cascades, linking gasotransmitter crosstalk to transcriptional stress-response programs [30]. Critically, these three molecules, melatonin, NO, and H_2_S, do not act redundantly but form a sequential amplification network in which each molecule expands the signaling output of the previous one, converging on ROS homeostasis, the AsA-GSH cycle, and ion channel regulation to confer tolerance to multiple stresses.

In addition, Chen et al. [35] reported that high temperatures trigger the production of H_2_S in seeds, primarily through enzymes such as *L*-cysteine Desulfhydrase (DES1) and *D*-cysteine Desulfhydrase (LCD). H_2_S significantly increased germination rate, as evidenced by experiments with H_2_S donors such as NaHS and GYY4137, resulting in increased germination under high-temperature stress. The underlying mechanism involves the H_2_S modulation of the nucleocytoplasmic partitioning of the COP1 protein, which is crucial for light and environmental stress responses. This modulation affects the degradation of the HY5 protein, a vital transcription factor involved in light signaling and stress response, subsequently influencing the expression of ABI5, a key regulator of seed germination and stress response. This complex interaction underscores the importance of H_2_S in plant development, particularly in adapting to environmental challenges such as high temperatures, highlighting its role in plant physiology and its potential applications in agriculture [35].

With a focus on rice plants, Gautam and coworkers focused on the effects of ethylene, NO, and H_2_S under temperature stress. Research has revealed that exposure to high temperatures (40 °C for 6 h per day for 15 days) significantly reduced biomass, photosynthesis, and leaf water status, but increased levels of oxidative stress markers, such as H_2_O_2_ and thiobarbituric acid reactive substances, in the leaves. The application of these signaling molecules increased the biomass, leaf water status, osmolytes, antioxidants, and photosynthesis in plants under both normal and high-temperature conditions. The effects were more pronounced with ethylene than with NO or H_2_S. Interestingly, the application of an H_2_S scavenger reversed the positive effects of ethylene or NO on photosynthesis under high-temperature stress, suggesting that H_2_S plays a role in ameliorating the effects of ethylene and NO. This study highlights the importance of H_2_S, along with ethylene and NO, in enhancing thermotolerance in plants and protecting photosynthesis under high-temperature stress [36].

To explore the challenges posed by heavy metal toxicity, Shivaraj et al. examined the intricate biosynthetic pathways of NO and H_2_S in plant systems, providing a detailed account of their production and cellular functions [37]. The interaction between NO and H_2_S can influence the expression of genes involved in the stress response. These genes encode antioxidant enzymes, metal chelators, and other proteins that help mitigate the toxic effects of heavy metals. One of the key protective roles of NO-H_2_S is the induction of antioxidant systems in plants. These systems help scavenge ROS generated by heavy metal stress, thereby reducing oxidative damage. Through these mechanisms, crosstalk between NO and H_2_S enhances plant tolerance to heavy metal stress. This can involve sequestering heavy metals, reducing their availability and toxicity, and repairing the damage they cause. Finally, NO and H_2_S help maintain cellular homeostasis under heavy-metal stress. They can regulate ion channels, influence cellular pH, and modulate other cellular processes disrupted by heavy metals [37].

## 4. The Use of NO and/or H_2_S Supplements in Agricultural Research

Agronomic applications of NO and H_2_S have evolved considerably from simple inorganic salts to increasingly sophisticated delivery strategies. Conventional donors such as sodium nitroprusside (SNP), sodium hydrosulfide (NaHS), and S-nitrosoglutathione (GSNO) have been extensively employed in experimental settings because of their low cost and ease of handling, but their short half-lives, photosensitivity, and corelease of potentially interfering byproducts impose significant limitations on their translational potential. More recently, the emergence of nanomaterial-based platforms has enabled the encapsulation or immobilization of these donors within carrier matrices that provide controlled, sustained, and environmentally responsive release profiles, substantially improving gasotransmitter bioavailability and reducing off-target effects. Table 2 and Table 3 (See below) provide a systematic overview of the main NO and H_2_S donor systems reported in the plant science literature, respectively, organized by supplement type, release mechanism, and documented physiological action, and serve as a reference framework for the comparative analysis developed in the following subsections.

### 4.1. Nanomaterials for Gasotransmitter Delivery

**Figure 5:**
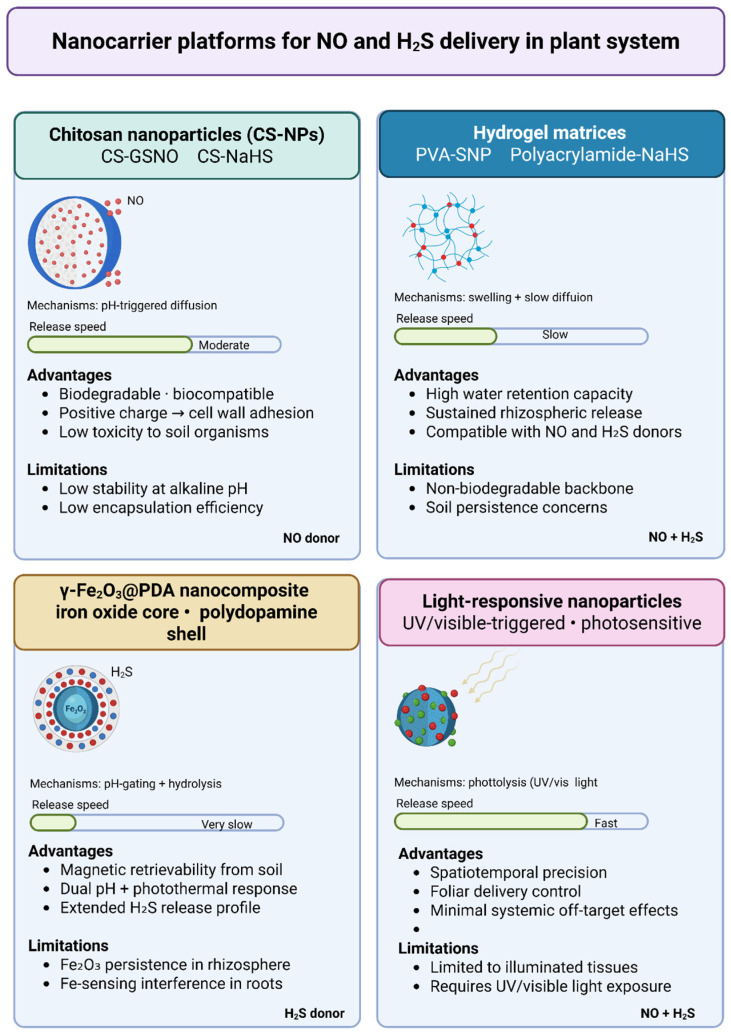
Nanocarrier platforms for controlled NO and H_2_S delivery in plant systems. Four classes of nanoplatforms are compared: chitosan nanoparticles (CS-NPs), which release NO donors via pH-triggered diffusion at apoplastic pH (5.0–6.5); hydrogel matrices, which provide slow sustained release of both NO and H_2_S donors through polymer swelling and diffusion; γ-Fe_2_O_3_@PDA nanocomposites, which exploit pH-gating of the polydopamine shell and slow hydrolysis of GYY4137 for very slow H_2_S release, with the added advantage of magnetic retrievability from soil; and light-responsive nanoparticles, which achieve rapid spatiotemporally controlled release of NO and H_2_S donors via photolysis upon UV or visible light exposure. For each platform, the release speed (bar length), key advantages (teal), and main limitations (coral) are indicated. Badge color indicates the gasotransmitter delivered: blue (NO), teal (H_2_S), and purple (NO + H_2_S). No single platform simultaneously optimizes release kinetics, biodegradability, tissue specificity, and ecological safety, underscoring the need for hybrid next-generation delivery systems.

The use of nanomaterials for the controlled release of NO and H_2_S represents a significant advance over conventional donors, offering sustained, environmentally responsive delivery profiles that substantially improve gasotransmitter bioavailability and reduce off-target effects [5,34,38,39]. Four principal nanoplatform classes have been reported in the plant science literature: chitosan-based nanoparticles (CS-NPs), hydrogel matrices, magnetic nanocomposites (γ-Fe_2_O_3_@PDA), and light-responsive photosensitive particles, each differing in release mechanism, cargo specificity, biodegradability, and agronomic suitability. A comparative overview of these platforms, including their release kinetics, key advantages, and main limitations, is presented in Figure 5. At the same time, the specific donors, application methods, and documented physiological effects are systematically compiled in Table 2 and Table 3 (See below). Critically, no single platform simultaneously optimizes release kinetics, biodegradability, tissue specificity, scalability, and ecological safety, a limitation that motivates the development of hybrid and sequentially triggered next-generation delivery systems.

#### Chemical Challenges of NO–H_2_S Co-Encapsulation

A critical gap in the current nanocarrier literature is the lack of true dual-gas delivery platforms capable of coreleasing NO and H_2_S in a coordinated, physiologically relevant manner from a single nanocarrier. The available platforms described above deliver either NO or H_2_S independently, and the few studies combining both gasotransmitters do so by sequentially or simultaneously applying separate donors rather than through integrated coencapsulation [39]. This distinction is not trivial: the synergistic effects of NO and H_2_S observed at the molecular level depend critically on the spatiotemporal overlap of both molecules at shared protein targets, and any delivery strategy that physically separates the two donors cannot reliably guarantee the concentration ratios required for cooperative PTM dynamics at the cysteine residue level.

Coencapsulation of both donors within a single nanocarrier faces substantial chemical incompatibilities that have not yet been resolved in plant systems. NO is a highly reactive radical that can oxidize thiol-containing H_2_S donors within the carrier matrix, potentially generating nitrosothiol byproducts before delivery to the plant tissue, thereby altering the intended signaling chemistry before release [29]. This reactivity is compounded by a pronounced kinetic mismatch between the available donors: NO donors such as GSNO operate on release timescales of hours, whereas H_2_S donors such as GYY4137 release their cargo over days to weeks, such that simple coencapsulation within a homogeneous matrix would inevitably produce temporally misaligned gasotransmitter pulses at the target tissue [39,40]. Spatially segregating the two donors within compartmentalized or Janus-type nanoarchitectures represents the most promising strategy to circumvent both chemical incompatibility and kinetic mismatch, allowing independent tuning of each donor release profile while maintaining their spatial proximity at the delivery site. Nevertheless, such systems have not yet been developed or validated in plant systems.

An alternative approach that avoids the coencapsulation problem entirely is the synthesis of NOSH-type hybrid molecules: small organic compounds that release both NO and H_2_S from a single chemical scaffold, thereby guaranteeing their stoichiometric co-release at the same site and time. A proof-of-concept for this strategy has been demonstrated in drought-stressed Medicago sativa [39]; however, nanoencapsulation of NOSH compounds to improve their stability, bioavailability, and controlled release under agronomic conditions remains entirely unexplored. Closing this technological gap, whether through Janus nanoarchitectures, stimuli-responsive compartmentalized carriers, or nanoencapsulated NOSH scaffolds, represents one of the most tractable and high-impact directions for the field.

### 4.2. Agronomic Applications of NO and H_2_S Donors

Conventional NO and H_2_S donors, including sodium nitroprusside (SNP), sodium hydrosulfide (NaHS), and S-nitrosoglutathione (GSNO), as well as their nanoencapsulated derivatives, have been extensively evaluated across a range of crop species, application routes (foliar, seed priming, and nutrient solution), and stress conditions, with documented benefits for antioxidant defense, photosynthetic performance, and postharvest quality. A systematic overview of these individual donor systems, organized by supplement type, release mechanism, and documented physiological action, is provided in Table 2 and Table 3. However, the agronomic potential of these molecules is most fully realized not through their independent application, but through their coordinated co-delivery, which exploits the biochemical crosstalk described in Section 2 and Section 3. The synergistic actions arising from combined NO–H_2_S treatments, and the experimental evidence supporting their cooperative roles across multiple stress scenarios, are analyzed in detail in Section 4.3.

**Table 2:** Overview of nitric oxide (NO) donors used in plant systems, including supplement type, delivery mechanism, physiological actions, and associated benefits.

Supplement Type	Application/Release	Action	Ref.
Sodium nitroprusside (SNP)	Foliar application	Mitigates water stress · improves growth and productivity · increases chlorophyll content and photosynthetic rate · activates antioxidant system · reduces lipid peroxidation.	[41]
SNP · NaNO_2_ · NH_4_NO_3_ · NaNO_3_ · NO gas	NO release in acidic environment · direct NO donation	Improves physiological and biochemical performance under Cu stress · reduces Cu toxicity via antioxidant enzyme activation and ion regulation	[42]
GSNO encapsulated in chitosan nanoparticles (CS-GSNO)	Controlled release via chitosan polymer matrix degradation	Gradual NO release · promotes photosynthesis, stomatal conductance, and recovery of water potential after water stress · enhances antioxidant and photoprotective responses	[43,44]
S-nitrosoglutathione (GSNO)	Slow and controlled NO release via spontaneous or light/metal-catalyzed decomposition	Alleviates water deficit · increases biomass, photosynthesis, relative water content, and antioxidant enzyme activity	[45]
Photosensitive nanoparticles with NO donor	NO release under UV/visible light irradiation (light-responsive systems)	Localized NO release enhances the response to abiotic stresses such as salinity	[46]
SNP (Sodium nitroprusside)	Chemical decomposition in aqueous solution · acts as a redox signaler and transcriptional modulator.	Increases firmness by modulating lignin and cellulose synthesis · activates phenylpropanoid pathway genes · enhances symbiosis with arbuscular mycorrhizal fungi · reduces cadmium bioavailability in soil	[47,48]
NO gas (fumigation, 10 μL L^−1^)	Direct gas release in a controlled environment	Reduces chilling injury and decay · increases firmness and vitamin C content · improves membrane stability · preserves volatile compounds · upregulates LOX/ADH/HPL/AAT pathways	[49]

**Table 3:** Overview of hydrogen sulfide (H_2_S) donors used in plant systems, including supplement type, delivery mechanism, physiological actions, and associated benefits.

Supplement Type	Application/Release	Action	Ref.
NaHS · Na_2_S (sodium hydrosulfide · sodium sulfide)	Rapid release of H_2_S in aqueous solution	Enhances antioxidant activity and redox balance · mitigates abiotic stresses (salinity, drought, heavy metals) · improves photosynthesis and delays senescence · regulates ion balance · interacts with NO signaling	[18,50]
NaHS + SNP (coapplication)	Direct release of H_2_S and NO; synergistic interaction between both gasotransmitters	Reduction in salt stress damage · enhancement of antioxidant activity and plant growth	[51]
γ-Fe_2_O_3_@PDA-GYY4137 nanocomposite	Slow and sustained release of H_2_S via GYY4137 hydrolysis, encapsulated in polydopamine (PDA) and magnetic iron oxide (γ-Fe_2_O_3_)	Stimulates salt stress tolerance · enhances plant growth · increases antioxidant enzyme activity · boosts nitrogen assimilation	[52]
Di(t-butanol)dithiophosphate phenyl thylamine (fBDPA) encapsulated in polylactic acid (PLA)	H_2_S released via dithiophosphate hydrolysis and PLA degradation, enabling sustained localized delivery	Increased radish yield (up to 141%) · improved germination · stress protection · stimulated root growth · localized, safe, and effective delivery	[53]
Nanoscale sulfur (nano-S)	Microbial conversion of elemental sulfur (S^0^) into H_2_S in the rhizosphere by sulfur-reducing bacteria	Enhances plant tolerance to abiotic stresses (salinity, drought, heavy metals), increases antioxidant activity, and upregulates defense-related gene expression.	[40]

### 4.3. Synergistic Actions and Crosstalk

The combined application of nitric oxide (NO) and hydrogen sulfide (H_2_S) donors has emerged as a promising agronomic strategy to mitigate multiple abiotic stresses, including heat, drought, salinity, and heavy metal toxicity, by preserving photosynthetic efficiency, strengthening antioxidant defenses, and sustaining plant productivity. Recent studies have demonstrated that sodium nitroprusside (SNP) and sodium hydrosulfide (NaHS) act synergistically to activate the ascorbate–glutathione (AsA–GSH) cycle, reduce oxidative markers such as H_2_O_2_ and malondialdehyde (MDA), and stabilize photosynthetic pigments and membrane integrity. The use of specific scavengers, cPTIO (2-(4-carboxyphenyl)-4,4,5,5-tetramethylimidazoline-1-oxyl-3-oxide, NO scavenger) and hypotaurine (H_2_S scavenger), consistently abolishes these protective effects, confirming a cooperative, interdependent role for both gasotransmitters in stress adaptation [3,38,54,55,56].

#### 4.3.1. Heat Stress

In wheat and tomato, the application of SNP and NaHS (via foliar or root treatments) has been shown to attenuate heat-induced decreases in photosynthesis by increasing the chlorophyll content, stomatal conductance, and photosystem II efficiency (Fv/Fm, maximum quantum yield of PSII). This protection is linked to the upregulation of the AsA–GSH cycle enzymes ascorbate peroxidase (APX), monodehydroascorbate reductase (MDAR), dehydroascorbate reductase (DHAR), and glutathione reductase (GR), and to an enhanced capacity for ROS detoxification [4,38].

#### 4.3.2. Drought Stress

Under water deficit conditions, exogenous NO increases the cellular AsA/GSH pool and reduces GSSG accumulation, whereas H_2_S enhances ABA-mediated stomatal regulation and osmotic adjustment. Their joint application leads to improved leaf water potential, reduced electrolyte leakage, and higher relative water content, ultimately improving biomass retention under drought [56].

#### 4.3.3. Salinity Stress

In salt-stressed wheat and rice, the SNP + NaHS treatments increased the AsA–GSH cycle, upregulated the expression of ion homeostasis genes (SOS1 and NHX1), and maintained K^+^/Na^+^ ratios, thereby increasing pigment stability, growth, and yield. Similar findings in cucumber and maize reveal cross-signaling that reprograms redox metabolism and photosynthetic carbon assimilation [56,57,58].

#### 4.3.4. Heavy Metal Stress

A combined supply of NO and H_2_S mitigates chromium- and cadmium-induced oxidative toxicity by reducing ROS levels, chelating free metal ions through thiol accumulation, and enhancing glutathione-dependent detoxification. This dual treatment restored chlorophyll synthesis, enzyme activity, and overall growth performance in several crops [59,60].

#### 4.3.5. Application Strategies

The experimental design involves different delivery routes, including foliar spraying, seed priming, or supplementation through nutrient solutions, depending on the crop species and the predominant abiotic stress. Validation controls are implemented by adding cPTIO and hypotaurine to confirm that these gasotransmitters specifically mediate the observed physiological effects. The practical markers used to evaluate treatment effectiveness include both short-term and long-term indicators: short-term improvements are characterized by reductions in H_2_O_2_ and MDA, alongside increased activities of antioxidant enzymes such as ascorbate peroxidase (APX) and glutathione reductase (GR), and improved photosynthetic efficiency measured by the maximum quantum yield of photosystem II (Fv/Fm). Long-term benefits include enhanced biomass accumulation, a relatively high SPAD index (an indicator of chlorophyll content), and increased grain yield, reflecting overall improved plant performance and stress resistance [3,4,5,54,55,56,57,58,59,60,61].

#### 4.3.6. Conflicting Evidence and Context-Dependent Outcomes

Although synergistic interactions between NO and H_2_S have been widely documented, an increasing body of evidence demonstrates that their crosstalk is not universally cooperative and that antagonistic, dose-dependent, and species-specific outcomes are equally well supported in the literature. Corpas et al. [9] conducted a critical analysis of available data. They concluded that the hierarchical relationship between these two gasotransmitters is not fixed but is determined by the plant organ, species, and experimental conditions under consideration. This conclusion was substantiated experimentally by [62], who demonstrated in *Solanum lycopersicum* under salt stress that NO precedes H_2_S in the signaling cascade, in direct contrast to the H_2_S-upstream hierarchy established for *A. thaliana* guard cells [32]. In the tomato system, exogenous NO stimulated the transcription of genes encoding H_2_S-synthesizing enzymes and elevated endogenous H_2_S levels. In contrast, exogenous H_2_S failed to reciprocally induce NO accumulation within equivalent time windows [62]. These species-specific reversals of signaling hierarchy indicate that mechanistic conclusions derived from one model system cannot be generalized across plant taxa without experimental validation.

Dose dependency constitutes a further source of inconsistency that is frequently underappreciated in studies that employ a single donor concentration. Both NO and H_2_S exhibit a concentration-dependent biphasic response: at submicromolar to low micromolar concentrations, they function as cytoprotective signaling molecules that activate antioxidant defenses and promote stress acclimation, whereas at elevated concentrations, they both transition to phytotoxic agents, NO, through the generation of reactive nitrogen species, including peroxynitrite (ONOO^−^) and nitroso-adducts at supraphysiological levels, and H_2_S through inhibition of cytochrome oxidase activity and disruption of sulfur homeostasis [9,50]. The concentration thresholds governing these transitions are species- and tissue-specific, such that a donor concentration that produces synergistic protection in wheat seedlings may induce oxidative injury in tomato fruit tissue under nominally identical experimental conditions.

The chemical properties of the donor compounds introduce an additional confounding variable that systematically limits cross-study comparisons. Fast-releasing donors such as NaHS generate a rapid, high-amplitude H_2_S pulse that activates acute stress-response cascades but may also saturate cellular persulfidation capacity, leading to off-target protein modifications that are not representative of endogenous H_2_S signaling. Slow-release donors such as GYY4137, by contrast, generate sustained low-level H_2_S concentrations that more closely approximate endogenous biosynthetic fluxes and produce qualitatively distinct transcriptional and proteomic responses [51]. Analogously, SNP releases NO concomitantly with cyanide and ferrous ions as decomposition byproducts, which independently modulate plant redox metabolism and enzyme activities, precluding unambiguous attribution of observed physiological effects to NO signaling per se [9].

Collectively, the conflicting outcomes documented in the literature indicate that the functional interaction between NO and H_2_S in plants is not a fixed synergistic partnership but rather an emergent property of the specific combination of plant species, organ, developmental stage, stress type, gasotransmitter concentration, and donor chemistry. Resolving these inconsistencies will require experimental designs that systematically vary each parameter independently and methodological advances, such as genetically encoded ratiometric biosensors capable of resolving real-time endogenous NO and H_2_S dynamics at subcellular resolution, approaches that remain largely absent from the current plant gasotransmitter literature.

### 4.4. Toward Sustainable Gasotransmitter-Based Agriculture

A dimension consistently overlooked in the gasotransmitter delivery literature is that soil microorganisms are major endogenous sources of both NO and H_2_S in the rhizosphere, and this microbial production occurs continuously and independently of exogenous donor applications. Denitrifying bacteria generate NO as an obligate intermediate of the stepwise reduction in nitrate (NO_3_^−^) to molecular nitrogen (N_2_) via the enzymes nitrite reductase (NirS/NirK) and nitric oxide reductase (Nor), and this microbially derived NO can diffuse into root tissues and modulate plant signaling at concentrations comparable to those applied exogenously [63]. Similarly, sulfate-reducing bacteria and sulfur-oxidizing microorganisms in agricultural soils continuously generate and consume H_2_S as part of the sulfur biogeochemical cycle, establishing a dynamic equilibrium of rhizospheric sulfide concentrations that respond to soil pH, redox potential, temperature, and organic matter availability [64].

Critically, the microbial H_2_S pool interacts bidirectionally with the plant: root exudates modulate the composition and activity of denitrifying and sulfur-cycling microbial communities, whereas microbially derived NO and H_2_S, in turn, influence root architecture, auxin signaling, and symbiotic associations with mycorrhizal fungi and rhizobia [64]. This bidirectional chemical dialog means that the interpretation of exogenous donor application experiments in soil-grown plants is inherently confounded by the background microbial gasotransmitter flux, a variable rarely measured or controlled in the current literature and that varies substantially across soil types, cropping systems, and climate conditions. Integrating microbiome-aware experimental designs, including the use of sterilized soils, gnotobiotic systems, or microbial community profiling alongside gasotransmitter measurements, represents an essential methodological advance for the field.

Beyond their role as endogenous gasotransmitter sources, rhizosphere microbial communities are also potential targets of exogenous NO and H_2_S donor applications, and this impact has received virtually no attention in the plant science literature. At low concentrations, NO functions as an interkingdom signaling molecule that regulates biofilm formation, quorum sensing, and nitrogen fixation activity in rhizobacteria, and exogenously applied NO donors at agronomic concentrations could plausibly modulate these processes in ways that are either beneficial or disruptive depending on the microbial community composition and the prevailing soil conditions [63,64]. In particular, arbuscular mycorrhizal fungi (AMF), which colonize the majority of terrestrial crop species and are critical for phosphorus acquisition and nitrogen cycling, are highly sensitive to changes in soil redox chemistry and gaseous signaling environments. However, no study has directly evaluated whether NO or H_2_S donor applications alter AMF colonization rates, hyphal network architecture, or nutrient transfer efficiency in crop plants.

Similarly, plant growth-promoting rhizobacteria (PGPR), such as Azospirillum, Bacillus, and Pseudomonas, which increase plant growth through nitrogen fixation, phosphorus solubilization, and phytohormone production, are known to be modulated by NO signaling in terms of colonization behavior and biofilm dynamics [63]. Exogenous H_2_S donors, by altering local soil sulfur chemistry and pH, could further shift the balance between sulfur-oxidizing and sulfur-reducing microbial guilds, with cascading effects on sulfur nutrient availability to plants. These interactions represent a critical knowledge gap: if exogenous gasotransmitter applications inadvertently suppress beneficial symbioses or alter nutrient cycling pathways, the agronomic benefits observed in controlled pot experiments may not translate, or even be counterproductive, under field conditions where complex plant‒microbiome interactions are fully active.

Despite substantial laboratory and greenhouse evidence supporting the agronomic potential of NO and H_2_S donors [38,55], the transition from controlled experimental conditions to open-field applications remains largely unrealized. A critical analysis of the barriers underlying this translational gap is essential for orienting future research toward strategies with genuine agronomic feasibility.

The most immediate physicochemical barrier is the chemical instability and toxicological profile of the most commonly used donors. While they are widely employed because of their low cost and ease of handling, SNPs simultaneously release NO, five cyanide ions (CN^−^), and iron (Fe^2+^) upon decomposition, which are byproducts that independently modulate plant proteome composition, enzyme activity, and ROS metabolism [65]. Its use as a selective NO donor is therefore debatable, and its use under open-field conditions is additionally constrained by UV/visible-light-driven photodegradation, which accelerates cyanide release under solar irradiation below 480 nm [65]. An additional, frequently overlooked constraint is the pH-dependent release of NO from several widely used inorganic donors. SNPs, NaNO_2_, and related nitrite-based compounds generate NO most efficiently under acidic conditions (pH < 6.5), as the protonation of nitrite to nitrous acid (HNO_2_) is required for non-enzymatic NO production. In neutral to alkaline soils, which constitute a substantial proportion of globally cultivated agricultural land, including calcareous and saline‒alkali soils, this protonation equilibrium shifts strongly against HNO_2_ formation, severely limiting effective NO delivery to the rhizosphere regardless of the applied donor concentration [9]. This pH dependency is rarely acknowledged in greenhouse studies conducted in acidified growth media. It represents a critical barrier to translating laboratory results to field conditions in neutral or alkaline cropping systems. Alternative donors with pH-independent release mechanisms, such as GSNO or diazeniumdiolate-based compounds, or encapsulation strategies that create localized acidic microenvironments around the nanocarrier, offer more practical solutions for deployment across the diverse soil pH ranges encountered in global agriculture. Similarly, NaHS dissociates nearly instantaneously in aqueous solution, generating a nonphysiological H_2_S pulse that bears little resemblance to the tightly regulated endogenous sulfide fluxes produced by DES1 enzymatic activity [9]. Under field conditions, these kinetic liabilities are further compounded by the wind-mediated dispersion of gaseous species, dilution by rainfall or irrigation water, and adsorption onto soil organic matter, all of which reduce effective plant exposure to a fraction of the nominal applied dose. Although encapsulation in nanocarriers partially addresses these limitations, the current cost of synthesis and quality control for materials such as CS-GSNO or γ-Fe_2_O_3_@PDA-GYY4137 composites remains prohibitive for broad-acre agricultural deployment [52].

A second barrier concerns the role of the soil microbiome in modulating the bioavailability of NO and H_2_S in the rhizosphere. Denitrifying microbial communities, whose composition and activity are governed by soil pH, temperature, water content, and organic carbon availability, actively transform exogenously applied nitrogen donors via nitrification and denitrification pathways, converting NO into N_2_O, N_2_, or NO_3_^−^ before plant roots can absorb them [63]. Analogously, sulfur-oxidizing bacteria present in most agricultural soils can rapidly oxidize H_2_S to sulfate or reduce it to polysulfides, substantially altering the chemical speciation and bioavailability of applied H_2_S donors [66]. Elevated soil temperatures, as predicted under accelerating climate change scenarios, are expected to amplify microbial metabolic rates, thereby accelerating gasotransmitter catabolism in the rhizosphere. Furthermore, irrigation practices introduce an additional variable: drip irrigation concentrates donor compounds in a restricted soil volume, potentially generating phytotoxic local concentrations, whereas flood or sprinkler irrigation dilutes and spreads donors over a larger area, increasing leaching losses.

A third barrier is regulatory. As noted in Section 4.1, no harmonized framework currently exists for the approval of gasotransmitter-based agricultural inputs, whether as conventional donors or nanoencapsulated formulations. There are no established maximum residue levels for SNP decomposition byproducts in edible crops, no standardized ecotoxicological testing for chronic low-level H_2_S donor exposure in soil ecosystems, and no clear regulatory pathway for combined NO/H_2_S codonor formulations [39]. Addressing these barriers will require both innovation in delivery chemistry, including enzyme-responsive donors that better mimic endogenous biosynthetic fluxes, and investment in multiseason, multisite field trials that capture the variability imposed by real agronomic conditions, combined with proactive engagement with regulatory bodies to define appropriate safety evaluation frameworks for this emerging class of crop inputs.

## 5. Questions and Perspectives

The gasotransmitters NO and H_2_S have emerged as critical signaling molecules that influence various aspects of plant life and physiological performance. However, the integrated mechanisms of NO and H_2_S, particularly under real agronomic or field conditions, remain poorly understood, with most studies focusing on their isolated effects and under controlled conditions [9]. This review highlights the synergistic interplay between NO and H_2_S, explores nanoapplication methods, and emphasizes their potential to increase plant resistance to abiotic and biotic stresses [3,4,5]. From the above, new questions and issues inevitably arise that merit exploration and further analysis, with the prospect of developing tangible technological proposals in the near future.

Some of the questions and perspectives the authors seek to highlight are:

**a.**  **What are the integrated mechanisms of the gasotransmitters NO and H_2_S under real agronomic or field conditions in different soils, climates, and plant species?**

There is a compelling practical reason to address the above question beyond routine studies conducted in controlled environments. An incomplete understanding of the molecular mechanisms underlying gasotransmitters in the agricultural field hinders the development of a solid theoretical basis for their application in crop production. The scarcity of studies at the hectare level [9,38] contrasts with the abundant evidence from controlled-condition studies, which highlight their stress-protective effects and significant promise as biostimulants.

For example, the balance between nitrosation and persulfidation influences the activity of many antioxidant and signaling enzymes [21,27,28], altering the plant response to ecological factors and affecting the absorption and assimilation of specific nutrients, as well as carbon metabolism. Whether persulfidation or nitrosation predominates likely hinges on intracellular NO and H_2_S levels, the prevailing cellular redoxtasis, and other multiple factors whose impact in the open field is much more complex than that observed in scientific studies. Similarly, stomatal activity, hormonal balance, and the synthesis of osmoprotectants are modulated by gasotransmitters [32,34] and by environmental factors whose effects vary depending on the scale of the growth environment.

The result of the interplay of gasotransmitters is a general state of stress tolerance induction (priming) in which it is difficult to assign one or another response to a specific gasotransmitter. Plant priming has been shown to induce durable physiological and epigenetic states [67] that increase crop resilience against stress. According to the above, it is expected that crop productivity and resilience will increase with the use of NO and H_2_S; however, no studies have been conducted on the commercial production of different crop species, plant genotypes, and climate conditions, nor on different grower-operating conditions. These studies have yet to confirm this expected gain.

**b.**  **How can advanced delivery systems, e.g., nanotechnology-based carriers, increase the synergistic potential of NO–H_2_S crosstalk?**

Advanced nanodelivery systems can prolong, localize, and modulate the synchronized release of NO and H_2_S [5,43,68], potentially converting their well-documented biochemical interplay into durable, field-relevant gains in crop stress tolerance and productivity. However, to the best of our knowledge, the following research gaps exist in this area: true dual-gas nanocarriers for crops are lacking. These dual nanocarriers must release NO and H_2_S in appropriate amounts and at the correct time to modulate crop physiological activities. Additionally, no information is available on the long-term soil persistence of support matrices (e.g., silica or nanogels) or their potential effects on the microbiome [39,45]. There is a significant gap in field-scale validation, as existing data are mainly from pot or growth-chamber experiments [38,50].

**c.**  **What are the potential ecological impacts of using gasotransmitters NO and H_2_S with an advanced nanodelivery system in agricultural practices?**

NO and H_2_S can reprogram plant metabolism at nanomolar levels [9,22], thereby increasing stress tolerance. Their volatility and short half-life have traditionally hampered commercial field use [9,65]. Advanced nanodelivery platforms enable slow, regulated release at lower application rates and may facilitate the co-delivery of both gases. Their use, however, requires careful analysis of the pros and cons in environmental terms, considering that evidence indicates that NO, H_2_S, and their metabolites appear to function not only as inter- and intraorgan signals but also as mediators of plant–plant or plant–microbiome interactions [63,64]. The authors of this review believe that such an analysis should include at least the following: (i) full life-cycle assessments integrating donor synthesis, field application and end-of-life nanocarrier fate [39]; (ii) a long-term mesocosm or hectare trials (at least three seasons) tracking soil health and biodiversity, carrier degradation and gas flux dynamics [39,45]; (iii) the thresholds for nontarget organisms such as pollinators and other beneficial arthropods and edaphic and aquatic biota under combined exposure to NO, H_2_S and the carrier [39,69]; and (iv) the impact on crop nutritional quality in terms of density and stoichiometric balances of essential minerals and phytochemicals with nutraceutical functions [1,5]. All the above methods require a large amount of information that is currently not fully available but is necessary for the future development of decision-support tools that consider soil type, climate data, and crop species or varieties to recommend doses, timings, and carriers.

On the other hand, other perspectives that the authors allow themselves to point out are as follows:

Combination of advanced delivery systems and field monitoring: The development of nanotechnology-based delivery systems for NO and H_2_S will increase the precision and efficiency of their application in agricultural systems. These systems aim to ensure the prolonged bioavailability of gasotransmitters while minimizing environmental losses and toxicity risks. On the other hand, for monitoring, portable NO/H_2_S electrochemical sensors and leaf-disk or petiole assays enable on-farm tracking of gasotransmitter status, facilitating adaptive management [17]. The above is relevant to the potential mid- or long-term persistence of carriers and gasotransmitters.

Optimization of application: Future research will focus on optimizing the modes, concentrations, and combinations of NO and H_2_S applications across different plant species and developmental stages to maximize their benefits in enhancing crop resilience and productivity.

Integration with other technologies: Integrating gasotransmitter research with other biotechnological and agronomic advancements, including genetic transformation, metabolic engineering, drones, robotics, and AI, could lead to the development of more resilient and sustainable agricultural systems that address global food security demands in the context of climate change [70].

**d.**  **How can omics technologies and genome editing tools advance our understanding of NO–H_2_S crosstalk in crops?**

The molecular complexity of NO–H_2_S signaling, characterized by competing post-translational modifications, hybrid reactive intermediates, and context-dependent synergistic or antagonistic outputs, demands analytical approaches capable of capturing these dynamics at a systems level. Omics platforms represent a transformative opportunity in this regard. Transcriptomic analyses using RNA-seq have begun to reveal the global gene expression networks activated by individual NO and H_2_S donors in model and crop species; however, studies employing combined gasotransmitter treatments under field-relevant stress conditions remain scarce [9]. Redox proteomics, particularly site-specific approaches such as the biotin-switch technique and iodoTMT-based quantification, enable the simultaneous identification of S-nitrosylated and persulfidated cysteine residues across the proteome, offering a direct window into the competitive PTM equilibrium described in Section 2.1 [21,27,71,72]. Integrating these datasets with metabolomic profiling of reactive sulfur species (RSS) and reactive nitrogen species (RNS) would allow the construction of comprehensive interaction maps linking gasotransmitter levels to downstream metabolic reprogramming. To date, no multi-omics study has captured the transcriptomic, proteomic, and metabolomic signatures of combined NO–H_2_S treatment across any crop species, thereby representing a critical knowledge gap.

Genome-editing technologies, particularly CRISPR-Cas9, offer complementary, highly precise tools for interrogating and engineering NO–H_2_S crosstalk in planta. Targeted loss-of-function editing of key biosynthetic genes, including nitrate reductase (NR), NOS-like enzymes, L-cysteine desulfhydrase (DES1 and LCD), and D-cysteine desulfhydrase (DDC), would generate defined gasotransmitter-deficient or gasotransmitter-enriched backgrounds in which the individual and combined contributions of NO and H_2_S to stress tolerance can be dissected with precision [73]. Furthermore, base editing and prime editing strategies could be used to introduce specific cysteine-to-alanine substitutions in key shared targets such as GAPDH (Cys156), RBOHD (Cys890), or OST1 (Cys137), allowing the functional consequences of blocking individual PTM sites to be assessed in vivo without disrupting the overall protein structure [21,32,74]. Beyond functional dissection, CRISPR-mediated upregulation of DES1 or LCD expression via transcriptional activators (CRISPRa) could serve as a breeding strategy to enhance endogenous H_2_S levels and optimize NO–H_2_S signaling ratios in elite crop varieties under climate stress [73]. The integration of multi-omics data with CRISPR-based functional genomics thus represents one of the most tractable and high-impact research frontiers for translating mechanistic knowledge of NO–H_2_S crosstalk into tangible crop improvement outcomes.

## Figures and Tables

**Table 1:** Shared molecular targets of NO and H_2_S in plant cells, their post-translational modifications (PTMs), mode of interaction, signaling context, and supporting references. Arrows indicate that the corresponding modification leads to the indicated functional effect.

Target/Pathway	NO Action (S-Nitrosylation)	H_2_S Action (Persulfidation)	Interaction Mode	Context	Ref.
GAPDH (GAPC1)	Inhibition at Cys156 → altered activity	Nuclear relocalization at Cys160	Competitive—opposing functional outcomes on the same protein	Redox stress · carbon metabolism	[21]
RBOHD (NADPH oxidase)	S-nitrosylation at Cys890 → inhibition of ROS production	Persulfidation → activation of NADPH oxidase · promotes ROS	Antagonistic—opposing effects on ROS burst	Guard cell ABA signaling	[21,27]
OST1/SnRK2.6	S-nitrosylation at Cys137 → kinase inhibition · fine-tunes ABA output	Candidate persulfidation target—functional outcome under investigation	NO-dominant in ABA response	Stomatal closure · ABA signaling	[21,32]
APX · GR (AsA–GSH cycle)	Upregulation of enzyme activity	Upregulation of enzyme activity	Synergistic—cooperative antioxidant defense	Oxidative stress · salinity · heat	[27]
MAPK cascades	Downstream activation → stress-response gene expression	Downstream activation → stress-response gene expression	Synergistic—convergent transcriptional output	Salinity · drought · heavy metals	[30]
K^+^/Na^+^ ion channels	Ion homeostasis regulation	Ion homeostasis regulation	Synergistic—cooperative ionic balance	Salinity stress	[28]
DES1 (H_2_S synthesis enzyme)	S-nitrosylation modulates DES1 activity → regulates endogenous H_2_S levels	Persulfidation activates DES1 → positive feedback on H_2_S production	Hierarchical—NO regulates H_2_S biosynthesis	Guard cell crosstalk	[21,32]

## Data Availability

No new data were created or analyzed in this study. Data sharing is not applicable.
